# Identification and Prevalence of Ixodid Ticks of Cattle in case of Haramaya Eastern Hararghe, Ethiopia

**DOI:** 10.1155/2021/8836547

**Published:** 2021-09-18

**Authors:** Anteneh Wondimu, Yehualashet Bayu

**Affiliations:** ^1^College of Agriculture and Natural Resource, Bonga University, Bonga, Ethiopia; ^2^College of Veterinary Medicine, Haramaya University, P.O. Box 138, Dire Dawa, Ethiopia

## Abstract

A cross-sectional study was conducted from December 2017 to April 2018 to determine the prevalence and identify major species of ixodid ticks of cattle and tick burden of different sex, age, breed, and body condition of cattle. Standard physical and direct stereomicroscopy techniques were employed for identification of tick species. During the study period, a total of 353 cattle were examined for presence of ticks and around 447 ticks were collected. The study showed that 34.3% cattle were infested with one or more tick species. The study reported different species of ticks in the order of their prevalence: *A. variegatum* (46.3%), *Rh. decoloratus* (20.1%), *A. cohaerens* (15.7%), *A. gemma* (11.9%), and *Rh. pulchellus* (6.04%), respectively. The prevalence of tick infestation between different risk factors such as sex, age, and body condition of cattle was statistically significant (*p* < 0.05). The overall male-to-female ratio of ticks was 2.29 : 1. Also, it was reported that, in *A. variegatum*, *A. cohaerens*, and *A. gemma*, the number of male exceeded that of female, but female number exceeded male number in case of *Rh. decoloratus*. The result also reported difference in attachment site preference, for example, *Amblyomma* genus was attached mostly to the scrotum/udder and axial and *Rh. pulchellus* was specified on the ear and perianal area, while *Rh. decoloratus* was non site selective. In conclusion, findings of this study suggest that ticks were the most important problems of cattle of the study areas. Therefore, the increasing threat of ticks warrants urgent strategic control including application of acaricides and creation of awareness among livestock owners about the veterinary importance of ticks for the integrated tick control.

## 1. Introduction

Livestock mainly cattle in Ethiopia represent the pillar of the economy and plays vital roles in generating income to farmers, ensuring food security, contributing to asset, social, cultural, and environmental values [1, 2]. Despite high livestock population and existing favorable environmental conditions, the current livestock output of the country is far below the expected potential. This is due to diseases which are a stumbling block to the potential of livestock industry [3]. Predominantly, parasitism by external and internal parasites in extensive and intensive production system is among the commonest problems [4]. Particularly, ectoparasites could create detrimental effects on their hosts through puncture, burrow, or attach onto the surface and cause discomfort, annoyance, weight loss, loss of condition, reduction in milk production, irritation of the skin, and predispose to infection [5, 6]. Moreover, they are the most important vectors for disease of veterinary importance such as protozoan, bacterial, viral, and rickettsial diseases [7].

Among the ectoparasites, ticks are well known for substantial effects in livestock production. Ticks are very common and extensively distributed in all agroecological zones especially in tropical and subtropical areas including Ethiopia [8]. They are harmful blood-sucking parasites and competent vectors of pathogens that affect both humans and animals [9, 10] and are usually considered to have more veterinary significance as a consequence of direct parasitism and disease transmission which negatively affect the health and productivity of livestock [11]. They pose serious economic losses to the farmer, the tanning industry, and the country as a whole [12]. In Ethiopia, tick and tick-borne diseases (TBDs) are economically very important diseases and cause substantial losses to the livestock economy, with an estimation loss of one million USD annually only through rejection of downgraded hides and skins attributed to tick damage [13].

Ticks that are most important to domestic animals' health in Africa include about seven genera and forty species [14]. In Ethiopia, isolated ticks belong to genus *Amblyomma*, *Rhipicephalus*, *Haemaphysalis*, *Rhipicephalus (Boophilus)*, and *Hyalomma* [15]. Several authors reported wide range of prevalence of tick infestation from different parts of Ethiopia, for example, 47.0% prevalence in cattle in Bishoftu town [16], 59.6% prevalence in Harar town [17], and 74.7% in Gondar town [18]. The seasonal variations may favor or hinder the development or activity of a tick species. *Amblyomma variegatum* and *Rhipicephalus (Boophilus) decoloratus* are considered to be the most prevalent and predominant tick species in higher rainfall season in Ethiopia [19]. From many reports, *A. variegatum* was the most common and widely distributed cattle tick in Ethiopia [20]. Dry environmental conditions are a serious danger to ticks, particularly to the larvae which are very susceptible to drying out fatally [15].

A study carried out six years before by Desalegn et al. [21] in the same study area reported that prevalence of tick infestation in cattle was found to be 25.2% and the most abundant species found were *Rh. decoloratus* (47.8%), *A. variegatum* (28.4%), *A. gemma* (12.5%), *Rh. pulchellus* (9.3%), and *Rhipicephalus evertsi evertsi* (2.02%) in Haramaya district. Another author reported 33.2% prevalence and three species of hard ticks, *A. variegatum*, *A. cohaerens*, *Rh. evertsi evertsi*, *Rh. pulchellus*, and *Rh. decoloratus* were recorded. This study also showed that *A. variegatum* was the most abundant of all tick species comprising 38.9% of the collected ticks in Haramaya district [22]. A very recent study conducted two years before showed higher prevalence of tick infestation in the study areas which was 73.9% in farms of Haramaya University. The identified ticks' genera were *Boophilus* (51.0%), *Amblyomma* (58.3%), *Hyalomma* (48.2%), and *Rhipicephalus* (53.1%) [23]. There is a wide range of difference in the prevalence of tick infestation reported by different authors and the previous study primarily carried out on Haramaya University farms which creates difficulty knowing the current status of ticks in Haramaya district. Hence, establishing the current status will help know tick fauna and their role as a vector agent, which is essential in understanding the epidemiology of such diseases and the designing of their appropriate control measures. Therefore, the present study was carried out with objective to estimate prevalence and related factors that contribute for the occurrence of tick infestation, Haramaya district, Ethiopia.

## 2. Materials and Methods

### 2.1. Study Area

The survey of identification of major ixodid ticks of cattle was conducted in Haramaya district, Ethiopia. Haramaya district is located 506 km east of Addis Ababa capital of Ethiopia. According to Haramaya district agricultural statistics information, the district has about 115,989 cattle, 88,144 sheep, 133,520 goats, 33,466 donkeys, 578 camels, and 137,545 chickens. The production system of the district is mixed type. Topographically, it is situated at an altitude of 1600–2100 m above sea level with the mean annual temperature and relative humidity of 18°C and 65%, respectively. The district receives an annual rainfall of approximately 870 mm with a range of 560–1260 mm and bimodal distribution pattern, picking in mid-April and mid-August. The vegetation that constitutes the available pasture lands in this area are predominantly native grasses and legumes interspersed with open acacia shrub land. Geographically, it is located at 9°24 N latitude and 42°01 E longitude, and ecologically, the area has 65% midland and 35% lowland zones [24].

### 2.2. Sample Size

Study cattle were sampled using simple random sampling technique at feeding burn from Haramaya University beef farm, and cattle were taken to Haramaya town veterinary clinic. The required sample size was determined by Thrusfeild [25] formula at 95% confidence interval:

(1) $$\begin{array}{c} n=\frac{1.96^{2}\left\{P_{\exp}\left(1-P_{\exp}\right)\right\}}{d^{2}}\;, \end{array}$$

where *n* = sample size, *P*_exp_ = expected prevalence (0.25), and *d* = desired precision (0.05).

According to Desalegn et al. [21], the prevalence of tick on cattle was 25.23% in Haramaya district. So, expected prevalence 0.25 was taken for this study. Using the above formula, the sample size is calculated to be 288, but to increase precision by 1.23 fold, a total of 353 animals were examined.

### 2.3. Study Design

A cross-sectional study was conducted from December 2017 to April 2018 to determine the prevalence and identify major species of ixodid ticks of cattle and tick burden of different sex, age, breed, and body condition of cattle. In this study, two study areas selected, namely, Haramaya University farm and Haramaya veterinary clinic, because of the easy access to cattle from different kebeles. Animals were randomly selected during the visit to Haramaya University farm using the lottery method, while Haramaya veterinary clinic animals were randomly sampled from cattle visiting for different diseases except for the case of ectoparasites spray. Different color dyes were used to mark sampled cattle to avoid repeated sampling in the clinic, while ear tags were used in Haramaya University farm. Age was determined using owner's information and dentition parameters. Age of the animals were determined as young (<1 year), adult (1–3 years), and old (>3 years) [26]. The body condition scores were classified as good, medium, and poor based on criteria set by Nicholson and Butterworth [26]. Poor body condition includes emaciated thin and starving body, where entire body is extremely thin and skeletal structure is prominently visible. Medium body condition is described as individual ribs noticeable but over fat cover is lacking. Good body condition includes moderately fat, very fleshy fat, and very obese. Bone structure is no longer noticeable.

### 2.4. Tick Collection and Identification

Sample collection was made after proper physical restraining of the animals; detectable ticks were carefully removed from the host for identification and count using hand. The whole body surface of the animals was examined for the presence or absence of ticks. Ticks were collected from different body parts of cattle such as head, ear, dewlap, scrotum, udder, perianal region, and the tail, which were kept separately in well-labeled bottle. During collection, ticks were removed manually from different attachment sites of the animal body by a rotating manner to retain their body parts for identification. Data collection format was used to register the data during tick collection, and proper labeling was made on universal bottles with a permanent marker. Code of animal, species, sex, age, body condition, and sites of attachment were included in the labeling. The collected ticks were placed into the universal bottle containing 70% ethanol for preservation and transported to Haramaya University Veterinary Parasitology Laboratory where ticks were counted and genus and species level was identified using a stereomicroscope, according to standard identification keys given by Walker et al. [15].

### 2.5. Statistical Analysis

All the data were entered on Microsoft Excel data base system, and they were analyzed by using Statistical Package for Social Sciences (SPSS) Version 20. Descriptive statistics was used to determine the prevalence of tick infestation in cattle. The overall prevalence of tick was determined by dividing the number of positive animals by total sample size and was expressed as percentage. The chi-square (*χ*2) test was used to point out the possible association of factors with the prevalence of tick infestations. Effects were reported as statistically significant in all cases if the value is less than 0.05 at 95% confidence interval (CI).

## 3. Results and Discussion

Ticks are one of the commonest ectoparasites distributed to different parts of Ethiopia, causing series economic losses in livestock production. The economic contribution of cattle is below the average for most countries including sub-Saharan Africa [3]. Among the contributing factors, ectoparasites including ticks causes serious economic loss to farmers, tanning industry, and the country as a whole through a direct and indirect effects on their hosts such as mortality of animals, decreased production, down grading, and rejection of skin and hide [4–6] and vectors for economically important animal diseases such as anaplasmosis, babesiosis, and *Cowdria ruminantium* [3, 7]. In this study, out of total 353 cattle examined, 121 (34.3%) cattle were infested with ixodid ticks. This finding is in agreement with the previous study of Kassa and Yalew [22] who reported 33.2% prevalence of ticks in the same study area and Desalegn et al. [21] who reported 25.23% prevalence. However, the finding disagrees with 61–89.4% prevalence reports in different parts of Ethiopia [23, 27–31]. This difference might be due to the difference in the agroclimatic condition of the study areas and season of sample collection. It was reported that tick activity can be influenced by rainfall, altitude, season, and atmospheric relative humidity [19]. In addition, large livestock population and herd size contribute to tick infestation as ticks can easily get access to hosts and complete their life cycle to continue rapidly, and poor veterinary service and less attention given for cattle management practice employed by herders might also pave way for the tick infestation.

In this study, prevalence was higher in male (39.4%) than in female cattle (28.6%) with statistical significance difference *p* < 0.05 (Table 1). This finding agrees with finding of Wasihun and Doda [28] who reported higher infestation in male animals compared to female. However, contrary to our finding, Abdeta et al. [18] and Kassa and Yalew [22] reported higher prevalence in female animals (68% and 18.8%) compared to male (82.06% and 14.23%), respectively. In another study, difference in infestation among sexes and the age groups of animals was not observed [23]. This variation may be related with female cattle kept in the house with proper management for dairy purposes while male cattle grazing on a field all day which can be exposed to tick infestation [28].

The proportion of tick infestation was higher in older than in adult and young age groups (Table 1). Difference between young and old was statistically significant (*p* ≤ 0.05). This finding was strengthened by the finding of Desalegn et al. [21] who reported infestation was higher in more than 3-year-old cattle than in less than 3-year-old cattle and also agrees with finding of different authors [18, 22, 23, 31] who reported higher proportion in adult cattle. Higher proportion may be due to long-distance movement of adult cattle to search for food which increases the chance of contact to tick and also low immunity in older animals.

In the current study, statistically significant difference was seen between good and poor body conditioned cattle *p* < 0.05 (Table 1). In consistent with our finding, Tamerat et al. [30], Abdeta et al. [18], and Wolde and Mohamed [32] reported high prevalence in poor body condition than moderate and good body conditioned cattle. This difference is because poor body conditioned animals had a low resistant to tick infestation and lack enough body energy to build resistance [28, 31].

The result of present study showed significant difference (*p* ≤ 0.05) (Table 1) in tick infestation between Haramaya University farm and Haramaya veterinary clinic; it is due to difference in the breeds, where sampled animals from farm was mostly *Bos taurus* breeds while those from the clinic was local breeds *Bos indicus.* Similarly, pure breeds and crossbreeds were reported being more innately resistant than *Bos taurus* breeds. The effects of ticks on indigenous cattle compared to exotic breeds were shown to be minimal [33, 34]. In addition to breed difference, the management system such as large livestock population and herd size contribute to tick infestation as ticks can easily get access to hosts and complete their life cycle to continue rapidly, and poor veterinary service and less attention given for cattle management practice employed by herders might also contribute for higher prevalence in samples from Haramaya veterinary clinic.

Overall, a total of 447 ticks were collected from 121 positive cattle. The most abundant species of tick was *A. variegatum* and the least one was *Rh. pulchellus*, as shown in Table 2. Prevalence of different species of ticks was as follows: *A. variegatum* (46.3%), *Rh. decoloratus* (20.1%), *A. cohaerens* (15.7%), *A. gemma* (11.9%), and *Rh. pulchellus* (6.04%), respectively. This finding agreed with Kassa and Yalew [22] who reported *A. variegatum* was the most abundant (38.87%), *Rh. decoloratus* (31.54%) was the second, and *Rh. pulchellus* was the least (6.64%). Several authors also strengthened the report that *A. variegatum* is the most common and widely distributed cattle tick in Ethiopia and African countries [19, 20, 23, 31, 35]. *Amblyomma variegatum* occurs in areas with a wide variety of climates ranging from highland Savannah to lowlands [15]. It is widespread and abundant in tick parasitizing cattle in the central highlands of Ethiopia as well as in the highland areas of the eastern parts of Ethiopia [8, 19]. *A. variegatum* has a great economic importance because it is an efficient vector of *Cowdria ruminantium*, the causative agent of cowdriosis or heartwater in cattle [36], the greatest damage to hides and skins because of its long mouth part, which renders the commodity valueless on world market if the infestation was high [37, 38], and also serves as a vector for emerging and re-emerging human and animal pathogenic bacteria including spotted fever group *Rickettsia*, *Coxiella burnetii*, and *Borrelia* spp [39]. In contrary, Tamerat et al. [30], Musa and Daba [39], and Belay [40] reported that *A. cohaerens* was found to be the most abundant tick species with a prevalence range of 36.4–50.5% in different parts of Ethiopia. This difference can be due to seasonal variation and agroecological difference.

The second abundant tick was *Rh. decoloratus*; the finding coincides with reports of different researchers. In Ethiopia, *Rh.* (*Boophilus*) *decoloratus* is the widely distributed tick species in different agroecological conditions and seasons of the country [22, 28, 41]. The distribution of *Rh. decoloratus* is similar to *A. variegatum* accompanied by wetter highlands and subhighlands receiving more than 800 mm rainfall [19].

The lowest proportion of *A. gemma* and *Rh. pulchellus* reported in the study area is consistent with Pegram et al. [19] who reported *A. gemma* and *Rh. pulchellus* are confined to semiarid areas and the lowland tick densities are usually greater than those in the highlands. It is also reported that *A. gemma* is an important tick of cattle and camels in eastern and southeastern parts of Ethiopia particularly in Afar, Somalia, and Harar and Eastern Tigray, Amhara, and SNNP regional states [41]. *A. gemma* was obviously associated with dry types of vegetation or semiarid rangelands and in lowland areas [42, 43]. *A. gemma* is widely distributed in woodland, bushland, and grassland in arid and semiarid area between the altitude 500–1750 meter above sea level and receiving 350–750 mm annual rainfall [33]. *Rh. pulchellus* has been reported as the most predominant tick species on camels in eastern Ethiopia, on small ruminants in eastern part of Ethiopia, and on cattle in Borena zone in Oromia region [44, 45]. *R. pulchellus* has been associated with a wide variety of pathogenic organisms affecting both animals and human, i.e., anaplasmosis, brucellosis, and anthrax [46]. This tick species transmits the protozoan *Theileria taurotragi* which causes benign bovine theileriosis. It has been implicated as a probable vector of Nairobi sheep disease that exists in north of Somali [47]. It can be a risk to humans because of its transmission of the bacterium *Rickettsia conorii*, causing tick typhus, and transmission of the virus of Crimean-Congo haemorrhagic fever. It may occur on some hosts in sufficient numbers to cause direct parasitic harm [15].

In *A. variegatum*, *A. cohaerens*, and *A. gemma*, the number of male exceeded that of female, but female number exceeded male number in case of *Rh. decoloratus*. Generally, the overall male-to-female ratio was 2.29 : 1 (Table 3). This finding agrees with Kassa and Yalew [22] study, earlier works of Solomon et al. [48], and Tamerat et al. [30] who reported the number of male ticks exceeded the female ticks. This is most probably attributed to the fact that fully engorged female ticks drop off to the ground to lay eggs while males tend to remain on the host up to several months later to continue feeding and mating with other females, as has been observed by Solomon et al. [48] and Tamiru and Abebaw [49], and the females of *Rh. decoloratus* outnumbered males in this study probably due to small size of male which may not be seen during collection [50].

With regard to predilection site for attachment, different tick species show different site preferences. *A. variegatum*, *A. cohaerens*, and *A. gemma* attach mostly to the scrotum/udder and axial but uncommon in other areas. *Rh. decoloratus* was distributed in all parts of cattle body except rare case on axial, scrotum, and udder, while *Rh. pulchellus* was frequently attached to perianal and inner parts of ear (Table 4). The finding is in line with the previous observation of Wasihun and Doda [28]. While *Rh. decoloratus* was found on the dewlap, udder, belly, head, neck, back, and scrotum, *Rh. pulchellus* showed high preference to the inside ear and anogenital region of the body. It was also reported earlier by Kassa and Yalew [22]. Site preference on the host depends on the convenience for attachment to get blood and protection to overcome the environment damage that hinders its existence and grooming activity of the host [51]. Genera with short hypostome, for example, Rhipicephalus species, usually attach to hairless areas such as under tail and anovulval area [52]. Generally, variety of factors such as host density, interaction between tick species, time and season, and inaccessibility for grooming determined the attachment site of the ticks on the skin [36].

## 4. Conclusion

The present study indicated 34.3% overall prevalence of tick in cattle in the study area. *A. variegatum* was found to be the most prevalent tick species identified. In addition, *Rh. decoloratus*, *A. cohaerens*, *A. gemma*, and *Rh. pulchellus* were also reported. Animal-related factors such as age, sex, breed, and body condition showed significant difference in tick infestation. Therefore, ticks are economically very important ectoparasites, which cause direct and indirect substantial economy losses to livestock sectors. Taking into account the effects of tick on livestock productivity, it is important to minimize the impact through effective tick control program which should be formulated and implemented at national and regional level based on the distribution pattern of ticks and factors responsible for their distribution. Moreover, attention should be given in creating community awareness about the impact of ticks, health care services, and management practices of cattle so as to control ticks which interns control problems that affect livestock production as a result of tick infestation.

## Figures and Tables

**Table 1:** Prevalence of tick infestation with respect to sex, age, body condition, and origin of cattle.

Risk factor		Examined cattle	Positive cattle	Prevalence (%)	OR	*p* value	95% CI	
							LB	UB
Sex	Male	178	71	39.4	1.3	0.025	1.06	2.6
	Female	175	50	28.6	0.8		0.7	0.9

Age	Young	39	4	10.26	0.2	0.002	0.06	0.54
	Adult	79	28	35.44	0.9	0.699	0.53	1.5
	Old ref^*∗∗*^							

Body condition	Moderate	132	51	38.63	0.7	0.159	0.37	1.2
	Good	145	30	20.69	0.4	0.002	0.2	0.7
	Poor as ref^*∗∗*^							

Origin	HU	140	56	40	0.7	0.04	0.6	1.1
	HC	213	65	30.5	1.1		0.9	1.4

OR, odd ratio; UB, upper boundary; LB, lower boundary; CI, confidence interval; HU, Haramaya University; and HC, Haramaya clinic. ^∗∗^Reference variable.

**Table 2:** Relative abundance of each species of tick in the study area.

Tick species	Frequency (N)	Percent (%)
*A. variegatum*	207	46.3
*A. cohaerens*	70	15.7
*A. gemma*	53	11.9
*Rh. decoloratus*	90	20.1
*R. pulchellus*	27	6.0
Total	447	100

**Table 3:** Identified tick species count with sex ratio in the study area.

Species	Sex of tick		Male-to-female ratio
	Male	Female	
*A. variegatum*	161	46	3.5 : 1
*A. cohaerens*	64	6	10.6 : 1
*A. gemma*	42	11	3.6 : 1
*Rh. decoloratus*	24	66	0.4 : 1
*Rh. pulchellus*	20	7	2.8 : 1
Total	311	136	2.29 : 1

**Table 4:** Number of tick in relation to attachment site on the animal body.

Site of attachment	Species of tick				
	*A. variegatum*	*A. cohaerens*	*B. gemma*	*Rh. decoloratus*	*R. pulchellus*
Scrotum/udder	161	45	53	9	0
Axial	68	25	0	3	0
Leg	7	0	0	5	0
Head	0	0	0	19	0
Ear and perennial	0	0	0	2	27
Flank	0	0	0	22	0
Neck	0	0	0	12	0
Belly	1	0	0	10	0
Back	0	0	0	8	0

## Data Availability

The data used to support the findings of this study are available from the corresponding author upon request.
